# The effects of cerebral pressure autoregulation status and CPP levels on cerebral metabolism in pediatric traumatic brain injury

**DOI:** 10.1007/s00701-024-06085-z

**Published:** 2024-04-24

**Authors:** Fartein Velle, Anders Lewén, Tim Howells, Anders Hånell, Pelle Nilsson, Per Enblad

**Affiliations:** Department of Medical Sciences, Section of Neurosurgery, Uppsala University Hospital, Uppsala University, SE 751 85 Uppsala, Sweden

**Keywords:** Traumatic brain injury, Children, Autoregulation, Optimal cerebral perfusion pressure, Cerebral microdialysis

## Abstract

**Background:**

Cerebral perfusion pressure (CPP) management in the developing child with traumatic brain injury (TBI) is challenging. The pressure reactivity index (PRx) may serve as marker of cerebral pressure autoregulation (CPA) and optimal CPP (CPPopt) may be assessed by identifying the CPP level with best (lowest) PRx. To evaluate the potential of CPPopt guided management in children with severe TBI, cerebral microdialysis (CMD) monitoring levels of lactate and the lactate/pyruvate ratio (LPR) (indicators of ischemia) were related to actual CPP levels, autoregulatory state (PRx) and deviations from CPPopt (ΔCPPopt).

**Methods:**

Retrospective study of 21 children ≤ 17 years with severe TBI who had both ICP and CMD monitoring were included. CPP, PRx, CPPopt and ΔCPPopt where calculated, dichotomized and compared with CMD lactate and lactate-pyruvate ratio.

**Results:**

Median age was 16 years (range 8–17) and median Glasgow coma scale motor score 5 (range 2–5). Both lactate (*p* = 0.010) and LPR (*p* =  < 0.001) were higher when CPP ≥ 70 mmHg than when CPP < 70. When PRx ≥ 0.1 both lactate and LPR were higher than when PRx < 0.1 (*p* =  < 0.001). LPR was lower (*p* = 0.012) when CPPopt ≥ 70 mmHg than when CPPopt < 70, but there were no differences in lactate levels. When ΔCPPopt > 10 both lactate (*p* = 0.026) and LPR (*p* = 0.002) were higher than when ΔCPPopt < –10.

**Conclusions:**

Increased levels of CMD lactate and LPR in children with severe TBI appears to be related to disturbed CPA (PRx). Increased lactate and LPR also seems to be associated with actual CPP levels ≥ 70 mmHg. However, higher lactate and LPR values were also seen when actual CPP was above CPPopt. Higher CPP appears harmful when CPP is above the upper limit of pressure autoregulation. The findings indicate that CPPopt guided CPP management may have potential in pediatric TBI.

## Introduction

Traumatic brain injury (TBI) still remains a major cause of death and morbidity in children worldwide [11]. Management strategies for pediatric TBI remains challenging and cannot always be extrapolated directly from those for adults. During the developmental transition from childhood through adolescence to adulthood anatomical and physiological changes occur that needs to be taken into account during neurointensive care (NIC) [15]. For example, the optimal cerebral perfusion pressure (CPP) threshold targets are difficult to assess since the physiological CPP varies with age. It is therefore important to gain further knowledge regarding pathophysiology of TBI in children in order to optimize NIC at an individual level [23]. An important factor that should be considered in regards to CPP management in children is cerebral pressure autoregulation (CPA) [34, 42]. CPA is a vascular response mechanism that maintains normal cerebral blood flow (CBF) despite changes in CPP. The pressure reactivity index (PRx) acts as a surrogate marker of CPA and is a continuously calculated correlation coefficient between slow fluctuations of mean arterial blood pressure (MAP) and intracranial pressure (ICP) [3, 8]. Negative PRx indicates functioning CPA, i.e. increasing MAP causes a responsive decrease in ICP due to cerebral vessel vasoconstriction and associated decrease in cerebral blood volume. Positive PRx indicates impaired CPA, i.e. increasing MAP causes passive dilatation of cerebral vasculature with increase of cerebral blood volume and hence increase of ICP. Impaired CPA is associated with worse outcome [10, 35]. The protective mechanism of CPA may be impaired due to TBI, increasing the brains vulnerability to hypo- and hyper-CPP levels, which in turn can lead to ischemia or edema [34, 42]. The optimal CPP (CPPopt) is the level at which the CPA works best and is calculated as the CPP level associated with the lowest PRx [2, 9, 37]. Instead of using fixed targets for CPP, CPA guided targets for CPP could be beneficial in NIC [2, 9, 20, 37], but have so far been studied to a lesser extent in children [5, 16, 19, 24, 27, 45]. In a previous study on children with severe TBI we found that impaired CPA was related to poor outcome, particularly in younger children and that CPP below CPPopt contributed significantly to the poor outcome [42].

A more direct indicator of the potential advantage of targeting CPPopt than clinical outcome would be to study the metabolic state of the brain in relation to CPA status and deviations from CPPopt. Cerebral microdialysis (CMD) is a minimally invasive technique to monitor chemical changes in the extracellular fluid that was introduced in NIC 1992 [31]. By using CMD the cerebral metabolic state of the brain can be assessed [4, 28]. Lactate and the lactate/pyruvate ratio (LPR) are markers of ischemia, where LPR is the main index of cellular redox state and the balance between oxidative and anaerobic metabolism [18]. Only a few studies have been published on CMD in children with severe TBI [6, 39, 40] and none of these studies have focused on CPA. In adult TBI studies, it has been shown that impaired PRx was associated with increased LPR levels [17] and that CPP values close to CPPopt were associated with lower LPR early after trauma and with lower glycerol later (marker of membrane degradation) [38].

The aims of the current study were to relate the metabolic state of the brain monitored by CMD to actual CPP levels, autoregulatory state (PRx) and deviations from CPPopt (ΔCPPopt) in children with severe TBI in order to evaluate the potential of individualized CPPopt guided management in those age groups.

## Materials and methods

### Patients and clinical data

Uppsala University Hospital serves the middle part of Sweden and the Department of Neurosurgery has a total catchment area of about 2 million people. Patients are admitted locally and from regional hospitals located within a distance of about 400 km.

All children ≤ 17 years, with severe TBI (defined as Glasgow Coma Scale motor score (GCSm) ≤ 5, assessed upon arrival) admitted to the NIC unit between 2007–2020 were eligible for this retrospective study. Among 82 eligible children, 61 had high resolution (100 Hz) monitoring data, 21 had CMD. Twenty-one children who had both high resolution monitoring data and CMD data were selected for this study. Demographics and clinical information were retrieved from patient records and Uppsala Traumatic Brain Injury register [30].

The following clinical variables were included: demographics, the Rotterdam CT score [25] of initial brain CT scan, GCSm on admission and departure from the NIC unit, barbiturate coma treatment (BCT) and/or decompressive craniectomy (DC), and outcome according to Glasgow outcome scale (GOS) [22] about 6 months after injury.

### Monitoring data collection and analysis

The ICP and arterial blood pressure waveform data were continuously recorded for each patient at a sampling rate of 100 Hz using the Odin software developed at Uppsala University and University of Edinburgh [20, 33]. All CPA measures were calculated retrospectively, i.e. the management was not guided by those parameters. PRx was calculated as 30 consecutive Pearson correlations of 10-s segments of high resolution ICP and MAP. This 5-min time-window moves forward in steps of 12 s so that five new values are produced per minute from which minute averages are calculated [7, 21]. CPPopt was calculated as the CPP associated with the minimum PRx over the last 4 h based on a quadratic model [2]. Deviations from CPPopt were denoted ΔCPPopt and calculated as the difference between actual CPP and CPPopt. For this study hourly means of CPP, PRx, CPPopt and ΔCPPopt were calculated and time-matched to the hourly collected CMD parameters lactate and LPR for each patient. ΔCPPopt is also presented as proportion of good monitoring time (%GMT) with ΔCPPopt < –10, ± 10 or > 10, respectively. GMT is the remaining monitoring time after removing time gaps with missing data due to e.g. surgery and radiology. Monitoring variables were analyzed from time of injury up to 10 days post-injury. Patients with less than 24 h of GMT within this time frame were excluded.

The CMD catheter was routinely placed in the in the right frontal lobe in non-lesioned tissue, through a separate burr-hole close to the intracranial monitoring device (see basal management below). A 71 High Cut-Off microdialysis catheter was used with a membrane length of 10 mm and a membrane cutoff of 100 kDa (M Dialysis AB, Stockholm, Sweden). The catheters were perfused by custom made sterile artificial cerebrospinal fluid (NaCl 147 mmol/L, KCl 2.7 mmol/L, CaCl_2_ 1.2 mmol/L, and MgCl_2_ 0.85 mmol/L supplemented with 1.5% human albumin (Perfusion Fluid CNS, M Dialysis AB) using a microinjection pump (106 MD Pump, M Dialysis AB) at a rate of 0.3 µL/min. The CMD samples were collected hourly and analyzed bedside using either the CMA600 or the ISCUSflex Microdialysis Analyzer (M Dialysis AB). Routine calibrations were performed. No specific treatments for CMD disturbances were used. Instead, the CMD monitoring was used as an early warning system for emerging treatable secondary insults.

### Neurointensive care management protocol

**Fig. 1:**
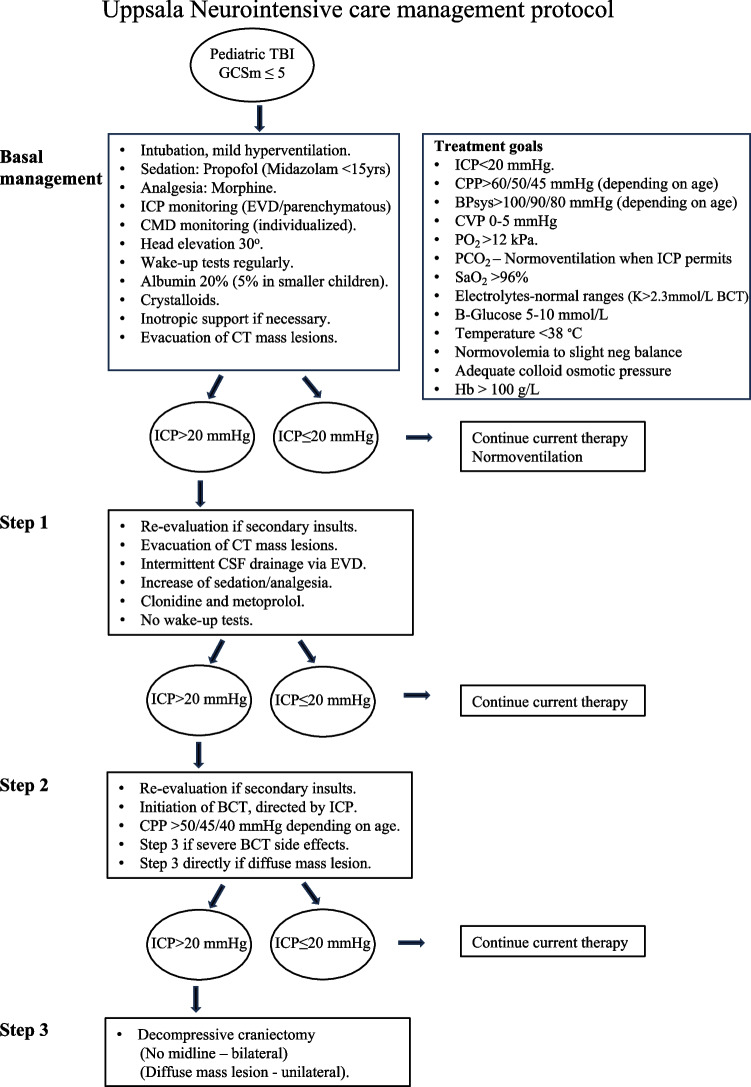
Uppsala Neurointensive care treatment goals and management protocol. TBI = traumatic brain injury; GCSm = Glasgow coma scale motor score; ICP = intracranial pressure; CPP = cerebral perfusion pressure; ABPsys = arterial blood pressure systolic; CVP = central venous pressure; HOB = head of bed elevation; EVD = external ventricular drain; CSF = cerebrospinal fluid; CMD = cerebral microdialysis; BCT = barbiturate coma treatment; CT = computed tomography

A standardized escalated ICP/CPP based management protocol is applied [12, 41], depicted in Fig. 1 and briefly described below. Treatment goals were ICP < 20 mmHg, CPP > 60/50/45 mmHg depending on age, systolic blood pressure > 100/90/80 mmHg depending on age, CVP 0–5 mmHg, body temperature < 38 ºC, normovolemia with adequate colloid osmotic pressure (infusions of Albumin 20% or Albumin 5% in small children, when needed), zero or slight negative water balance, electrolytes within normal ranges.

*Basal management *- Patients with GCSm ≤ 5 are intubated, and ICP and CMD (decided by the responsible neurosurgeon) are monitored. Sedation with propofol (midazolam in children < 15 years) and morphine as analgesic. ICP is monitored preferably with an intraventricular catheter (Smiths Medical®), to allow cerebrospinal fluid drainage when needed, or an intraparenchymal probe (Codman®) if the ventricles are small/compressed. Mild hyperventilation (pCO_2_ 30–34 mmHg/4.0–4.5 kPa) is applied initially and gradually changed to normoventilation as soon as ICP permits. Hypotension is treated with volume substitution (crystalloids and Albumin), and dobutamine or noradrenaline if required. Neurological state is assessed regularly by wake-up tests. In cases of high ICP, blood pressure is not artificially increased above normal levels to achieve an adequate CPP due to concerns about the risk for development of secondary brain edema in brain tissue with impaired blood brain barrier and/or deranged CVA [13]. Spontaneously high CPP levels are not actively lowered unless the elevated CPP is related to increased ICP. If ICP is increased despite the basal management a new CT scan is performed to rule out the presence of a mass lesion requiring surgical removal before escalation of management to the next level. *Step 1*—No further wake-up tests are performed, and more sedatives and analgesics are given. Infusions of α2-agonist and β1-antagonist are administered, but usually not in small children. If these measures do not decrease ICP, management is escalated further. *Step 2* – BCT [41, 43] is initiated provided that CT shows no significant mass lesion. In cases showing a diffuse mass lesion with shift of the midline, a decompressive hemicraniectomy is performed instead (Step 3). Thiopental is used as a mono-sedative. The lowest bolus and infusion doses needed to decrease ICP is administrated without intention of obtaining burst suppression on the electroencephalogram. CPP of > 50/45/40 mmHg is considered sufficient depending on age. When adequate doses of thiopental do not reduce ICP below 20 mmHg or BCT is not tolerated by the patient due to side effects the next management step is initiated. Due to concerns about rebound edema, osmo-therapy in the form of daily scheduled mannitol or hypertonic (3%) saline infusions is not used except for mannitol boluses in case of herniation. *Step 3*—Decompressive craniectomy is performed [44]. If there is diffuse swelling without midline shift, a bifrontotemporal decompressive craniectomy with duraplasty is performed with sparing of a bone ridge in the midline. When there is a diffuse focal mass lesion with midline shift, a large hemicraniectomy with duraplasty is performed instead.

### Statistical methods

**Fig. 2:**
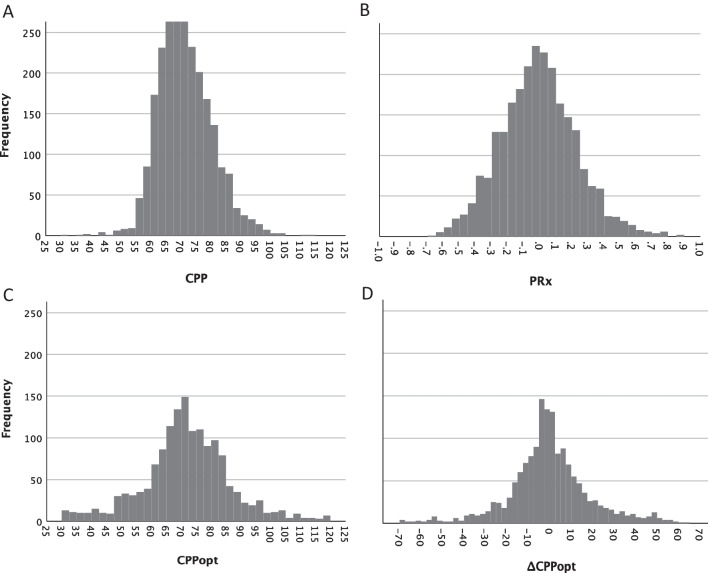
Histograms presenting the distribution of hourly averaged monitoring data (time-matched to CMD data): CPP (**A**), PRx (**B**), CPPopt (**C**) and ΔCPPopt (**D**)

All data were transferred to SPSS v 27 (IBM) for statistical analysis. Histograms were used to visualize the distribution of CPP, PRx, CPPopt and ΔCPPopt. The time-matched hourly means of continuous monitoring variables and CMD parameters were presented as mean (95% CI) for the whole studied monitoring period. Based on the histogram frequency distribution of CPP and CPPopt (Fig. 2), a cut-off at 70 mmHg was applied for dichotomization in order to compare results with CMD lactate and LPR. PRx was also dichotomized, with a cut-off at 0.1 according to the histogram distribution (Fig. 2) as well as to previous CPA studies using PRx in children with TBI [34, 42]. Non-parametric statistics were used, Mann-Whitney U test for dichotomized comparison and Kruskal-Wallis test with Bonferroni correction for trichotomized ΔCPPopt comparison. The limited cohort size of patients inhibited us from proceeding with multiple logistic analyses. Differences were considered statistically significant if *p* < 0.05.

## Results

### Patient characteristics, management and outcome

Patient characteristics, clinical management and outcome are presented in Table 1. The study included a total of 21 children with median age 16 years (range 8–17), median GCSm 5 (range 2–5) at admission and median Rotterdam CT classification 4 (range 3–5) of the first brain scan. All children were mechanically ventilated and had intracranial pressure monitoring; intraparenchymal probe (Codman®) in 13 children and intraventricular catheter (Smiths Medical®) in 14 (both in 6). Seven children received one, two or three of the following interventions: barbiturate coma treatment (n = 5), decompressive craniectomy (n = 5) and hematoma evacuation (n = 3). Median GCSm was 6 (range 1–6) at discharge from NIC and median GOS was 4 (range 1–5, 13 favorable (GOS 4–5) and 8 unfavorable (GOS 1–3)) six months after trauma. One child died.

**Table 1:** Patient characteristics, clinical management and outcome

Patients, n	21
Age (years), median (range)	16 (8–17)
Sex-male, n (%)	12 (57)
Cause of injury	
Motor vehicle occupant, n (%)	7 (33)
Pedestrian, n (%)	4 (19)
Sport, cyclist, fall, n (%)	9 (43)
Violence, n (%)	1 (0.5)
GCSm adm., median (range)	5 (2–5)
Rotterdam score, median (range)	4 (3–5)
Score 3, n (%)	7 (33)
Score 4, n (%)	8 (38)
Score 5, n (%)	6 (29)
External ventricular drain, n (%)	14 (67)
Parenchymal ICP monitor, n (%)	13 (62)
BCT, n (%)	5 (24)
DC, n (%)	5 (24)
Hematoma evacuation, n (%)	3 (14)
GCSm NIC dep., median (range)	6 (1–6)
GOS 6 months, median (range)	4 (1–5)

GCSm = Glasgow Coma Scale motor score at admission (adm) and departure (dep);

BCT = Barbiturate coma treatment;

DC = Decompressive craniectomy;

GOS = Glasgow outcome scale score

### CPP, PRx, CPPopt and ΔCPPopt

The distribution of monitoring data for the entire studied monitoring period is presented as frequency histograms in Fig. 2 and as mean values (95% CI) in Table 2. Median duration from trauma to start of intracranial pressure monitoring and CMD monitoring was 14.5 h (range 6–91). The median number of hours of contributing data from each patient was 121 h (range 24–228). Mean CPP for all children was 71.4 mmHg (95% CI 71.1 to 71.8 mmHg) and mean CPPopt 72.0 mmHg (95% CI 71.1 to 72.9 mmHg). Mean PRx for all children was –0.01 (95% CI –0.02 to 0.00). For all children the mean %GMT with ΔCPPopt < –10 was 32.6% (95% CI 25.9% to 39.5%), ΔCPPopt ± 10 42.0% (95% CI 37.0% to 47.1%) and ΔCPPopt > 10 25.1% (95% CI 20.3% to 29.9%), respectively.

**Table 2:** Mean monitoring values for the entire monitoring period

CPP, mm Hg	71.4 (71.1 to 71.8)
PRx	–0.01 (–0.02 to 0.00)
CPPopt, mm Hg	72.0 (71.1 to 72.9)
ΔCPPopt < –10, %GMT	32.6 (25.9 to 39.5)
ΔCPPopt ± 10, %GMT	42.0 (37.0 to 47.1)
ΔCPPopt > 10, %GMT	25.1 (20.3 to 29.9)

Values are presented as the mean (95% CI) for CPP, PRx, CPPopt, ΔCPPopt < –10 (%GMT), ΔCPPopt ± 10 (%GMT) and ΔCPPopt > 10 (%GMT)

### CPP, PRx, CPPopt and ΔCPPopt in relation to cerebral microdialysis

**Fig. 3:**
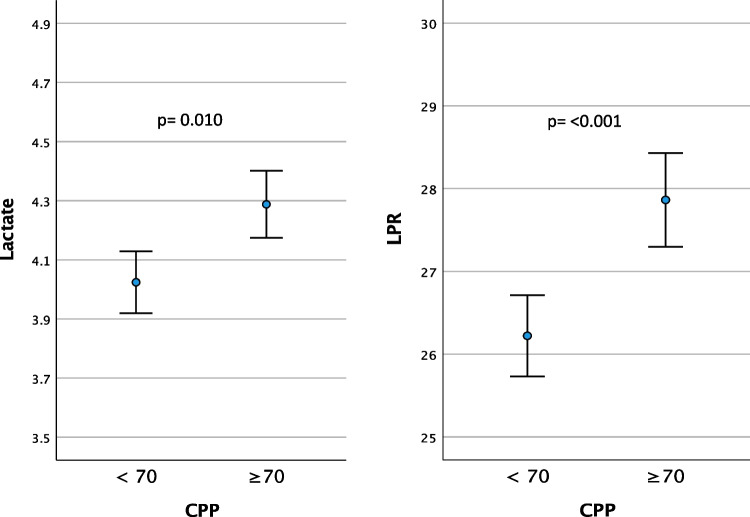
Lactate (mM) and LPR mean values (95% CI) presented by dichotomized CPP

**Fig. 4:**
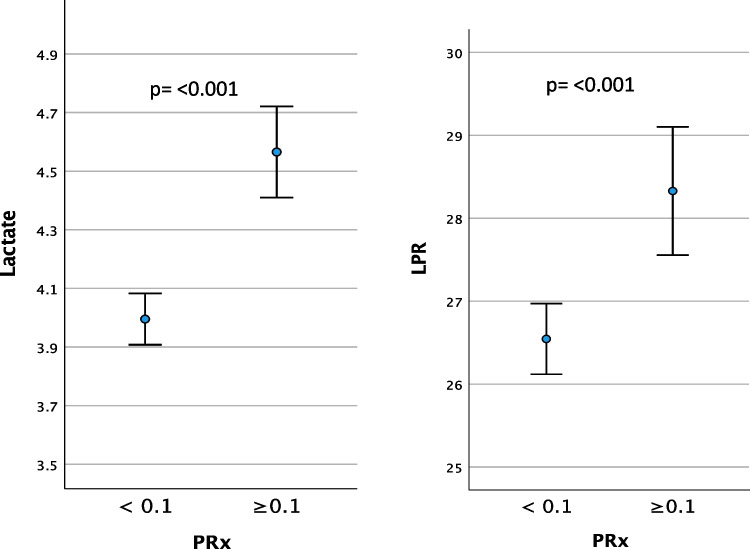
Lactate (mM) and LPR mean values (95% CI) presented by dichotomized PRx

**Fig. 5:**
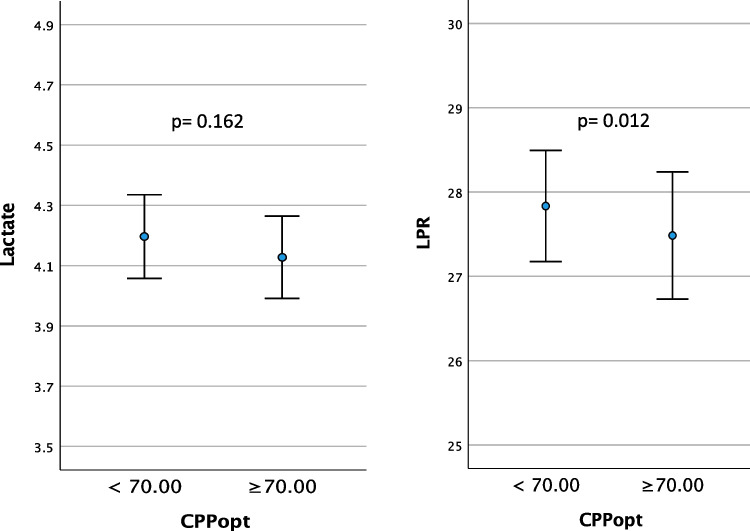
Lactate (mM) and LPR mean values (95% CI) presented by dichotomized CPPopt

The hourly lactate (mM) and LPR mean values (95% CI) are presented in relation to corresponding dichotomized hourly mean values of CPP, PRx and CPPopt in Table 3 and Figs. 3, 4, and 5. The time matched cerebral monitoring and CMD data showed significantly higher values in both lactate (4.29 mM [95% CI 4.17 to 4.40]) (p = 0.010) and LPR (27.86 [95% CI 27.30 to 28.43]) (p =  < 0.001) when CPP ≥ 70 mmHg as compared to lactate (4.02 mM [95% CI 3.92 to 4.13]) and LPR (26.22 [95% CI 25.73 to 26.71]) when CPP < 70 mmHg, respectively (Fig. 3. and Table 3). When PRx ≥ 0.1 both lactate (4.57 mM [95% CI 4.41 to 4.72]) (p =  < 0.001) and LPR (28.33 [95% CI 27.55 to 29.10]) (*p* =  < 0.001) were significantly higher than lactate (4.00 mM [95% CI 3.91 to 4.08]) and LPR (26.54 [95% CI 26.12 to 26.97]) when PRx < 0.1 (Fig. 4 and Table 3). CPPopt showed no significant differences in lactate levels between CPPopt < 70 and ≥ 70 mm Hg (4.13 mM [95% CI 3.99 to 4.26] vs 4.20 [95% CI 4.06 to 4.34]; p = 0.162). LPR was significantly lower (27.48 [95% CI 26.73 to 28.24]) (p = 0.012) when CPPopt ≥ 70 mmHg than LPR (27.83 [95% CI 27.17 to 28.49]) when CPPopt < 70 mmHg (Fig. 5).

**Table 3:** Mean values of cerebral microdialysis lactate and lactate-pyruvate ratio (LPR) for the monitoring period by dichotomized CPP, PRx, CPPopt and ΔCPPopt

	Lactate, mM	LPR
CPP < 70, mm Hg	4.02 (3.92 to 4.13)	26.22 (25.73 to 26.71)
CPP ≥ 70, mm Hg	4.29 (4.17 to 4.40)	27.86 (27.30 to 28.43)
*p*	**0.010**	** < 0.001**
PRx < 0.1	4.00 (3.91 to 4.08)	26.54 (26.12 to 26.97)
PRx ≥ 0.1	4.57 (4.41 to 4.72)	28.33 (27.55 to 29.10)
*p*	** < 0.001**	** < 0.001**
CPPopt < 70, mm Hg	4.20 (4.06 to 4.34)	27.83 (27.17 to 28.49)
CPPopt ≥ 70, mm Hg	4.13 (3.99 to 4.26)	27.48 (26.73 to 28.24)
*p*	0.162	**0.012**
ΔCPPopt < –10 ^a^	4.10 (3.90 to 4.30)	26.90 (25.77 to 28.04)
*p* (a vs b)	1.00	0.060
*p* (a vs c)	**0.026**	**0.002**
ΔCPPopt ± 10 ^b^	4.08 (3.94 to 4.22)	27.60 (26.91 to 28.29)
*p* (b vs a)	1.00	0.060
*p* (b vs c)	**0.003**	0.248
ΔCPPopt > 10 ^c^	4.39 (4.20 to 4.58)	28.47 (27.45 to 29.48)
*p* (c vs a)	**0.026**	**0.002**
*p* (c vs b)	**0.003**	0.248

Lactate (mM) and LPR mean values (95% CI) presented by dichotomized CPP, PRx and CPPopt, respectively, and by ΔCPPopt, ΔCPPopt < –10, ΔCPPopt ± 10 and ΔCPPopt > 10 (%GMT)

**Fig. 6:**
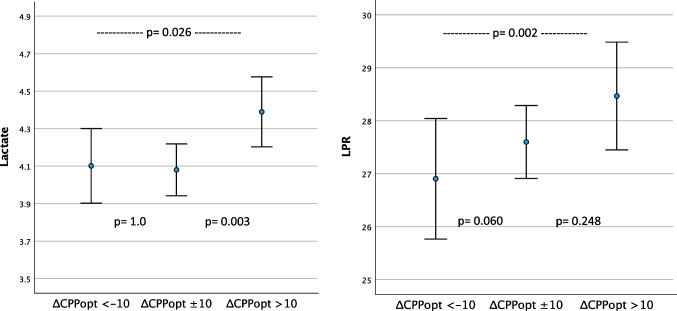
Lactate (mM) and LPR mean values (95% CI) presented by ΔCPPopt < –10, ΔCPPopt ± 10 and ΔCPPopt > 10

The CPP deviation from CPPopt denoted ΔCPPopt and the relation to CMD are presented in Table 3 and Fig. 6. There were significantly higher lactate (4.39 mM [95% CI 4.20 to 4.58]) and higher LPR (28.47 [95% CI 27.45 to 29.48]) during the periods of GMT when ΔCPPopt > 10 as compared to lactate (4.10 mM [95% CI 3.90 to 4.30]) (p = 0.026) and LPR (26.90 [95% CI 25.77 to 28.04]) (*p* = 0.002) during the periods of GMT when ΔCPPopt < –10.

## Discussion

In an earlier study of pediatric TBI we found that impaired CPA was related to poor outcome in all children, and in younger children actual CPP below CPPopt was also related to poor outcome [42]. In this retrospective study of 21 children with severe TBI the actual CPP levels, the integrity of CPA (PRx), and deviations from CPPopt (ΔCPPopt) were related to the metabolic redox state of the brain as assessed using CMD. Both lactate and LPR were significantly higher when CPP and PRx were higher, although the levels of lactate and LPR did not reach very high levels when compared to estimated normal values in adults [32]. The results give additional support for the role of PRx as a surrogate marker of CPA and that impaired CPA is negative for the brain. Furthermore, the results also indicate that higher CPP is unfavorable for the brain. A negative influence of low CPP was not seen but it is important to emphasize that very low CPP rarely occurred. When discussing the influence of CPP levels on cerebral metabolism it is also important to consider CPPopt and the impact of deviations from CPPopt. It is possible that the observed relation between the actual CPP levels and the microdialysis levels to some extent may be explained by deviations between actual CPP and CPPopt.

CPPopt appeared relatively high in this study. There is no clear explanation for that. CPP was also relatively high, much higher than our implemented lowest CPP threshold. One possible explanation for the observed high CPP may be that an upper CPP threshold to treat never has been implemented. Consequently, low CPP is intensively treated while high CPP is rarely treated. The relatively high CPP may have contributed to the high CPPopt, although the exact mechanisms are unclear.

No significant lactate and LPR differences were seen between CPPopt ≥ 70 or < 70 mmHg, which was expected and in line with our earlier findings that CPPopt was not related to outcome [42]. Interestingly, when ΔCPPopt was > 10 both lactate and LPR were significantly higher. It is therefore possible that this partly may be the reason behind why CPP was found to be associated with higher lactate and LPR. A possible mechanism may be that hyperperfusion occurs when actual CPP is above the upper limit of pressure autoregulation, which may lead to aggravation of the brain injury [13]. An unexpected finding was that LPR was not higher when ΔCPPopt < –10. The reason for that is unclear, but the limited number of children included and the observed relatively high mean CPP may be possible explanations.

In our earlier larger study deviations from CPPopt did not seem to influence outcome significantly when we looked at all included children while a larger proportion of GMT with ΔCPPopt < –10 in smaller children was associated with less favorable outcome [42]. These discrepancies may have several explanations. Fewer younger children were included in this study [42] and it is possible that younger children are more sensitive to CPP below CPPopt. Furthermore, even if the proportion of GMT with ΔCPPopt > 10 was similar in the two studies the degree of deviation from CPPopt may have been different. Finally, the comparison between the two studies needs to be cautious and it is therefore important to emphasize that clinical outcome and cerebral metabolism are different outcome variables and one cannot anticipate for sure that those are completely interrelated as discussed below.

One way of evaluating the clinical significance of disturbed CPA and the potential of using CPPopt for guidance of CPP treatment is to study those measures in relation to clinical outcome. Clinical outcome may however be influence by other factors than pathophysiology and management during NIC, e.g. rehabilitation efforts, social network and socioeconomic factors. A complementary way of evaluating CPA and CPPopt is to use a surrogate end point such as the chemistry of the brain which may be monitored by using CMD. CMD provides a possibility to analyze the chemical content of the interstitial fluid in the brain, i.e. it enables measurements of for example metabolites, excitotoxic amino acids and biomarkers. We chose to analyze lactate and LPR in this study to obtain information about the cerebral metabolism and the intracellular redox state of the brain [31]. Lactate and foremost the lactate/pyruvate ratio (LPR) are the most robust biochemical markers of ischemia reflecting secondary brain injury [14, 36]. In this study the extent of LPR elevations related to high PRx was quite modest, which differed compared to the results found in the large adult TBI study by Guilfoyle and coll [17]. One can speculate that this difference may depend on differences in the patient materials, e.g. type of injury, and in the management but also on age. Normal/abnormal ranges have not been established in the pediatric population and the assumption that the ranges are similar as in adults is uncertain [32]. Earlier studies in adults with TBI have also used CMD to assess the safe lower limit of CPP suggesting that management might be individualized [29]. This is a potential application of CMD in pediatric TBI also, although we could not identify any critical thresholds of CPP in this study, possibly because CPP was never critically low.

The major limitations of this study are the limited number of children included and that the average age of the children might be too high to generalize the results to children of all ages. Unfortunately, the limited number of patients also made multivariate statistical analysis impossible and direct causality is difficult to evaluate. Nevertheless, this is to our knowledge the first study evaluating the potential relevance of using information of CPA and CPPopt in NIC in children with severe TBI by relating those parameters to lactate and LPR in microdialysates from the brain.

## Conclusion

Overall, this study showed that higher levels of CMD lactate and LPR in children with severe TBI were related to disturbed CPA (PRx). Actual CPP levels ≥ 70 mmHg was associated with increased lactate and LPR. However, higher lactate and LPR values were also seen when actual CPP was above CPPopt and such situations may have contributed to the observed general effect of high actual CPP levels. It is likely that higher CPP not always per se is harmful but rather when CPP is above the upper limit of pressure autoregulation. The findings indicate that CPPopt guided CPP management may have potential, but further studies are required. Hopefully, larger multicenter studies like KidsBrainIT [26] and STARSHIP [1] will clarify this further.

## Data Availability

Data are available upon reasonable request.
